# Galectin-9 treatment is cytotoxic for B cell lymphoma by disrupting autophagy

**DOI:** 10.3389/fphar.2025.1601235

**Published:** 2025-06-26

**Authors:** Lisanne Koll, Harm Jan Lourens, Glenn Marsman, Stan de Haan, Toshiro Niki, Gerwin A. Huls, Edwin Bremer, Valerie R. Wiersma

**Affiliations:** ^1^ Department of Hematology, University Medical Center Groningen, University of Groningen, Groningen, Netherlands; ^2^ Department of Immunology, Kagawa University, Takamatsu, Kagawa, Japan

**Keywords:** galectin-9 (Gal-9), B cell lymphoma, cell death, autophagy, chemoresistance

## Abstract

**Introduction:**

The main cause of death for patients with non-Hodgkin lymphoma (NHL) remains therapy resistant relapses. Chemoresistance is commonly associated with apoptosis defects and upregulated autophagy. Therefore, novel therapeutic options that do not rely on apoptosis and target autophagy would be of interest to treat NHL. An agent that may fulfill these requirements is the glycan-binding protein Galectin-9 (Gal-9).

**Methods:**

A panel of B cell lymphoma NHL cell lines, including diffuse large B cell lymphoma (DLBCL), mantle cell lymphoma (MCL), Burkitt’s lymphoma (BL), and (chemoresistant) follicular lymphoma (FL), were treated with Gal-9 after which cell counts and cell viability were determined. Basal mRNA and protein expression levels were respectively determined by RTqPCR and western blot. The impact of Gal-9 treatment on the autophagy pathway was determined using lysotracker, Cyto-ID and western blot (targeting LAMP2, p62, LC3B-I/LC3B-II).

**Results:**

Treatment with Gal-9 reduced total cell counts and cell viability of various DLBCL, MCL, BL and FL cell lines. Gal-9-induced cell death was associated with the inhibition of autophagy, as demonstrated by the accumulation of LC3B-II and p62. In addition, Gal-9-sensitive cells expressed lower basal protein levels of LC3B-I as compared to cells that responded less to this lectin. Furthermore, Gal-9 was cytotoxic for chemoresistant Sc-1 cells (Sc-1-RES), which were even more sensitive toward Gal-9 treatment than the parental cells (Sc-1-PAR).

**Conclusion:**

Gal-9 is a potent inducer of B cell lymphoma cell dead by inhibiting the proper execution of autophagy.

## Introduction

Each year, over 2,000 patients are diagnosed with non-Hodgkin lymphoma (NHL) in the Netherlands [kanker.nl], and 545,000 worldwide (Chu et al., 2023). Despite increased survival rates in the past decades [LLS.org], the main cause of death for patients with NHL remains refractory disease and therapy resistant relapses. Specifically, for diffuse large B cell lymphoma (DLBCL), the most prevalent type of NHL (20%–50% of total), therapy resistance to the first line of treatment R-CHOP (rituximab, cyclophosphamide, doxorubicin hydrochloride (hydroxydaunorubicin), vincristine sulfate (Oncovin), and prednisone) is seen in one-third of the patients (Qualls et al., 2025). This resistance is commonly associated with aberrations in the apoptosis pathway (Bernal-Mizrachi et al., 2006; Johnson et al., 1993; Diepstraten et al., 2023), and the upregulation of autophagy (Mandhair et al., 2024), the latter being a survival pathway in which superfluous and damaged cellular components are degraded and recycled in a process that depends on the fusion of autophagosomes with lysosomes (Folkerts et al., 2019). Therefore, therapeutic targeting of autophagy instead of apoptosis may be of interest to eradicate NHL cells.

An agent that fulfills above requirements is Galectin-9 (Gal-9). Gal-9 is a member of the galectin family of glycan-binding proteins, and belongs to the group of ‘tandem-repeat’ galectins (Wiersma et al., 2013). These lectins bind to specific glycans using their two carbohydrate recognition domains (CRDs) that are connected by an inter-domain linker. We and others have previously demonstrated that Gal-9 is cytotoxic for various types of human cancer, including colon carcinoma (Wiersma et al., 2015), melanoma (Wiersma et al., 2012), and leukemia (Choukrani et al., 2023; Kuroda et al., 2010). Interestingly, Gal-9 also eradicated cancer cells resistant to conventional therapies, like cytarabine-resistant acute myeloid leukemia (AML) (Choukrani et al., 2023), imatinib-resistant chronic myelogenous leukemia (Kuroda et al., 2010), and apoptosis-resistant colon cancer (Wiersma et al., 2015). This induction of cell death by Gal-9 did not rely on apoptosis but on the impairment of autophagy execution (Wiersma et al., 2015; Choukrani et al., 2023). Therefore, we hypothesized that Gal-9 is also a promising option to induce cell death in (therapy-resistant) NHL.

Indeed, treatment of NHL, focused on B cell lymphoma in this study, induced cell death in the majority of tested DLBCL, mantle cell lymphoma (MCL), Burkitt’s Lymphoma (BL), and Follicular Lymphoma (FL) cell lines. Gal-9 also induced cell death in a chemoresistant FL cell line. Mechanistically, Gal-9 disrupted the execution of autophagy, whereby Gal-9 sensitivity associated with basal expression levels of LC3B-I. Thus, Gal-9 is a potent inducer of cell death in (chemoresistant) B cell lymphoma by inhibiting the proper execution of autophagy.

## Materials and methods

### Galectin-9

The Galectin-9 (Gal-9) protein used in this paper was produced as previously described (Nishi et al., 2005). If not specified otherwise, ‘Gal-9’ refers to the recombinant form of Gal-9 with a truncated linker, also known as Gal-9 (0). Gal-9(S) is a recombinant form of the natural Gal-9 isoform with the short linker (only used in Supplementary Figures S2A–B) which was provided by prof. Niki.

### Cell lines and healthy B cells

#### Commercial cell lines

All cell lines were originally purchased from established cell line banks, being ATCC or DSMZ. For nomenclature the names as recommended by Cellosaurus were used. This study included various Diffuse Large B cell Lymphoma (DLBCL), mantle cell lymphoma (MCL), follicular lymphoma (FL), and Burkitt’s Lymphoma (BL) cell lines. DLBCL: OCI-Ly3, DOHH2, SU-DHL-2, SU-DHL-4, SU-DHL-5, SU-DHL-6, SU-DHL-10, WSU-DLCL-2, Ri-1 and U-2932. MCL: REC-1, HBL-2, JeKo-1, Granta519 and UPN1. FL: Sc-1 and WFU-FSCCL. BL: Ramos and Daudi. All cell lines were cultured at 37°C in a humidified CO_2_ atmosphere using RPMI + 20% FBS (Gibco RPMI Medium 1,640, Catalog #52400025, ThermoFisher Scientific, Waltham, MA, United States and Sigma Aldrich, F7524, St. Louis, MO, United States). Of note, all cell lines were regularly STR-profiled and tested for *mycoplasma*.

#### Chemoresistant cell line panel

The Sc-1 cell line was made chemoresistant by culturing under gradually increasing doses of chemotherapy. Specifically, Sc-1 cells were cultured with vincristine (VNC, Hospital’s pharmacy), starting with a dose of 250 pM, eventually reaching a dose of 10 nM after 9 months. Subsequently, cells were cultured under constant pressure of 10 nM VNC plus an increasing dose of Doxorubicin (DOX, Hospital’s pharmacy), starting with 10 nM, eventually reaching a dose of 60 nM after 3 months. This VNC and DOX double-resistant cell line was called ‘Sc-1-RES’ and cultured under a constant pressure of 10 nM VNC plus 60 nM DOX. The parental Sc-1 cell line was cultured alongside the chemoresistant cells during the whole procedure to prevent differences caused by passage number, and called ‘Sc-1-PAR’. Of note, after obtaining the Sc-1-PAR and Sc-1-RES cell line panel, their Sc-1 STR-profile was confirmed and cells were tested *mycoplasma* free.

#### Healthy B cells

Healthy B cells were isolated from buffycoats (Sanquin, the Netherlands; under agreement number NVT0465). In brief, peripheral blood mononuclear cells (PBMCs’) were isolated using Lymphoprep™, following manufacturers recommendations (Stemcell technologies, Catalog # 18061). Subsequently, cells were pre-incubated with 100 μL FcR-blocking agent (Miltenyi, Catalog # 130–059-901, Bergisch Gladback, Rhineland, Germany) and incubated for 1 h at 4°C with anti-CD3-PE (20 μL, Clone: MEM-57, Immunotools, Catalog # 21270034, Friesoythe, Germany), anti-CD56-PE (20 μL, Clone: B-A19, Immunotools, Catalog # 21810564, Friesoythe, Germany), and anti-CD14-PE (15 μL, Clone: HCD14, Biolegend, Catalog # 325606, California, United States) to obtain B cells by negative selection. The cells were subsequently washed twice with 15 mL PBS and resuspended in PBS. Cells were then subjected to MACS-mediated cell sort, using anti-PE MACS beads (Catalog # 130–048-801, Miltenyi Biotec, Bergisch Gladback, Rhineland, Germany), following manufacturers recommendations. To evaluate purity, cells were stained with anti-CD19-PE (Clone LT19, Immunotools, Catalog # 21270194), before and after sorting. Analysis was performed on a BD Accuri C6 flow cytometer (BD Biosciences) and accessory C6 Plus analysis software.

### Cell death assays: cell counts and MTS assay

#### Cell counts

Cells were plated at 50.000 cells/well in a 48-wells plate in a final volume of 200 μL RPMI + 20% FBS. Gal-9 was added to the wells at the indicated concentrations and incubated for 24 h. In case of the lactose and sucrose experiments, 40 mM of each sugar was added to the wells before adding Gal-9 (Sigma-Aldrich, L3625 and G7021, St. Louis, MO, United States). Subsequently, cells were harvested and analyzed using a flow cytometer, either the BD Accuri C6 flow cytometer (see above) or the Cytoflex (Beckman Coulter, Brea, CA, United States). The counts of the cells (cells/μL) within the viable single cell gate (based on FSC/SCC and FSC/FSC-H) were used and cytotoxicity was calculated as (treated/untreated) x 100%. The reliability of this method was demonstrated with OCI-ly3 using propidium iodide (PI) (P3566, Invitrogen, ThermoFisher Scientific, Waltham, MA, United States), a dye that stains leaky cells. Indeed, the cells in the viable gate were PI negative, and Gal-9 treatment increased the amount of PI-positive cells (Supplementary Figure S1A).

#### MTS assay

The experimental setup was similar as for the cell count assays, but the incubation time was extended to 72 h. After 72 h, MTS (CellTiter 96^®^ AQueous One Solution Cell Proliferation, G3580, Promega, Madison, WI, United States) was added (7.5% v/v) and incubated until sufficient color development (OD-untreated ≥ 1). The read-out was performed at 490 nM (Multiskan SkyHigh Microplate Spectrophotometer, ThermoFisher Scientific Waltham, MA, United States) and each experimental OD490 was corrected by the OD490 of the ‘dead control’ (7.5% v/v ‘dead mix’, consisting of 10% Triton-X in 70% ethanol). Cell viability was calculated as percentage of the untreated control (treated/untreated*100%).

### Autophagy assays: Cyto-ID and lysotracker

OCI-Ly3 cells were plated at a density of 50.000 cells/well in a 48-wells plate (200 µL medium) and incubated with Gal-9 (300 nM) for 5 h. Six-wells were pooled and divided over 3 tubes; unstained, lysotracker (LysoTracker^®^ Red DND-99, 1 μM, Life Technologies, L-7528, Carlsbad, California, United States), and cyto-ID (1:1,000, ENZ-51031–0050, Enzo Lifesciences, Inc., New York, United States). After incubating 30 min at 37°C, cells were washed and imaged using the EVOS Cell Imaging System (EVOS-FL, Thermo Scientific, Waltham, MA, United States). Each experiment was performed for three independent times.

### Western blot

#### Sample preparation autophagy

Cells were plated at a density of 1 million/2 mL in a 12-wells plate in RPMI + 20% FBS (two wells per condition). To determine the impact of Gal-9 on the autophagy pathway, cells were incubated with 300 nM Gal-9 for 24 h. For autophagic flux measurements, cells were incubated with 50 µM chloroquine (CQ, positive control of the LC3B Antibody Kit for Autophagy, Invitrogen™, L10382, Carlsbad, CA, United States) for 6 h. Cells were harvested (two wells pooled) and washed with PBS before lysis in self-prepared lysis buffer (50 mM Tris, 2 mM EDTA, 2 mM EGTA, 150 mM NaCl, 0.1% SDS, 1% NP-40 substitute) containing 1 µM Na_3_VO_4_ (Sigma, 450,243, St. Louis, MO, United States) and protease inhibitor cocktail (Sigmafast; Sigma Aldrich, S8830, St. Louis, MO, United States). For the analysis of basal expression levels of autophagy proteins, 5 million cells of each cell line were washed with PBS (3x) and lysed in 100 µL of lysisbuffer. The total protein concentration was determined using the Bradford assay (Pierce™ Coomassie (Bradford) Protein Assay Kit, #23200, Thermo Scientific, Waltham, MA, United States) and 20 µg total protein was mixed with loading buffer containing β-mercaptoethanol as reducing agent. Of note, each experiment was repeated for five independent times for the autophagy analysis, and in duplicate for the basal expression level analysis of the cell line panel.

#### Western blot

After boiling the samples, they were loaded onto self-poured SDS-PAGE gels with a 15% polyacrylamide concentration (40% Acrylamide/Bis Solution, 37.5:1 #1610148, Biorad, Hercules, California, United States). The blotting process was performed using the Trans-Blot^®^ Turbo™ Transfer System (Biorad, Hercules, California, United States) and accessory Trans-Blot Turbo Transfer Packs with PVDF membranes (Biorad, #1704274, Hercules, California, United States), after which the membranes were blocked with 5% (w/v) milk powder/TBST. Membranes were cut and subsequently incubated with primary antibodies against LC3B (LC3B Antibody Kit for Autophagy, Invitrogen™, L10382, Carlsbad, CA, United States, 1:1,000), SQSTM1/p62 (SantaCruz, sc-28359, California, United States, 1:200), or LAMP2-HRP (H4B4, Santacruz, sc-18822 HRP, California, United States, 1:200) for overnight at 4°C on a rollerbank. The next day, membranes were washed with TBST (3x) and incubated with appropriate secondary HRP-conjugated antibodies (Dako, p0217, p0260, Santa Clara, United States, 1:2000) for 1 h at room temperature. After extensive washing with TBST (≥3x), blots were developed using chemiluminescent substrate (SuperSignal West Dura, Thermo Scientific, Life Technologies, 34,075, Waltham, MA, United States) and imaged using the ChemiDoc MP system (Bio-Rad, Hercules, California, United States). A loading control staining was performed on the p62-blot, by incubating with a directly HRP-conjugated anti-β-actin antibody (AC-15, ab49900, Abcam, Cambridge, United Kingdom, 1:10.000). In addition, a ponceau S staining was performed to double check for protein loading (Ponceau S solution, P7170-1, Sigma-Aldrich, St. Louis, MO, United States). Of note, all five independent experiments were subjected to Western blot.

#### Densitometry

Quantification of detected proteins was performed using the ImageJ tool for gel analysis. Each value was corrected for protein loading based on the density of the β-actin band. Of note, since different cell lines express different levels of β-actin, the correction was only performed within cell lines (untreated, Gal-9, CQ of the same cell line) and not between cell lines. All five independent experiments were quantified, and subsequently analyzed as a whole. Specifically, the factor change between the treatment condition (Gal-9 or CQ) and the untreated control was calculated based on the average of the untreated control of all experiments. Hence, the factor of the untreated control is 1 on average, but does show the variation between the five independent experiments. For treatment conditions a factor above 1 means an increase in protein levels, whereas a factor below 1 means a reduction in protein levels. To calculate the autophagic flux, the β-actin-corrected LC3B-II levels were used, whereby the value of the untreated control was subtracted from the CQ-treated condition. The delta values of all five independent experiments are depicted in the graph.

### RTqPCR

*Sample preparation:* mRNA was isolated from all B cell lymphoma cell lines (5 million cells) using the mRNA isolation kit (Qiagen RNeasy plus mini kit #74134) following manufacturers recommendations. mRNA yield and purity was determined with the above mentioned MultiSkan Sky device using the μDrop™ plate (ThermoFisher Scientific, #N12391, Waltham, MA, United States). The obtained mRNA was converted into cDNA using the iScript™ cDNA Synthesis Kit (Biorad; #1708891, Hercules, California, United States). For each cell line three independent samples were taken.

*RTqPCR:* cDNA (5 ng per condition) was mixed with SYBRgreen (Biorad, #1725274, Hercules, California, United States) following manufacturers recommendations and the following primers were used:

*LC3B* (for: TGC​GGG​CTG​AGG​AGA​TAC​AA, Rev: TCT​TTG​TTC​GAA​GGT​GCG​GC),

*SQSTM1* (for: GTG​AAG​GCC​TAC​CTT​CTG​GG, Rev: CGT​CCT​CAT​CGC​GGT​AGT​G),

*ATG5* (For: TGG​GAT​TGC​AAA​ATG​ATT​TGA​CC, Rev: TCC​TAG​TGT​GTG​CAA​CTG​TCC),

*LAMP1* (For: ATG​TGT​TAG​TGG​CAC​CCA​GG, Rev: TGT​TCA​CAG​CGT​GTC​TCT​CC).

*LAMP2* (For: TGG​CTC​CGT​TTT​CAG​CAT​TG, Rev: TGT​CAT​CAT​CCA​GCG​AAC​ACT).

*RPL27* (For: TCC​GGA​CGC​AAA​GCT​GTC​ATC​G. Rev: TCT​TGC​CCA​TGG​CAG​CTG​TCA​C).

The RTqPCR reaction was performed using the C1000 Touch Thermal Cycler (Biorad C1000, CFX384 Real-Time System) qPCR program: 3 min 95°C, (5 s 95°C, 30 s 58°C → 40 times), 3 s 65°C, increase till 95°C (in steps of 0,5°C of 3 s each).

### Statistical analysis

All graphs with multiple measurements show the average + SD. Statistical differences between the levels of autophagy proteins (untreated versus Gal-9 treated) were tested with the Mann-Whitney test. The statistical differences in autophagic flux between the 3 cell lines was tested with a Kruskal–Wallis test with Dunn’s multiple comparison test. Significant correlation between protein expression and Gal-9 sensitivity was tested using linear regression analysis. The statistical differences between the basal expression levels of autophagy proteins between Sc-1-PAR and Sc-1-RES were determined by a t-test. To correct for inter-experimental differences, all values were normalized towards the PAR value. All statistical analysis were performed using Graphpad Prism (version 8.02 or version 9.1.0). p-values are indicated as: *p < 0.05, **p < 0.01, ***p < 0.001, and ns = not significant.

## Results

### Galectin-9 is cytotoxic for B cell lymphoma cells

**FIGURE 1:**
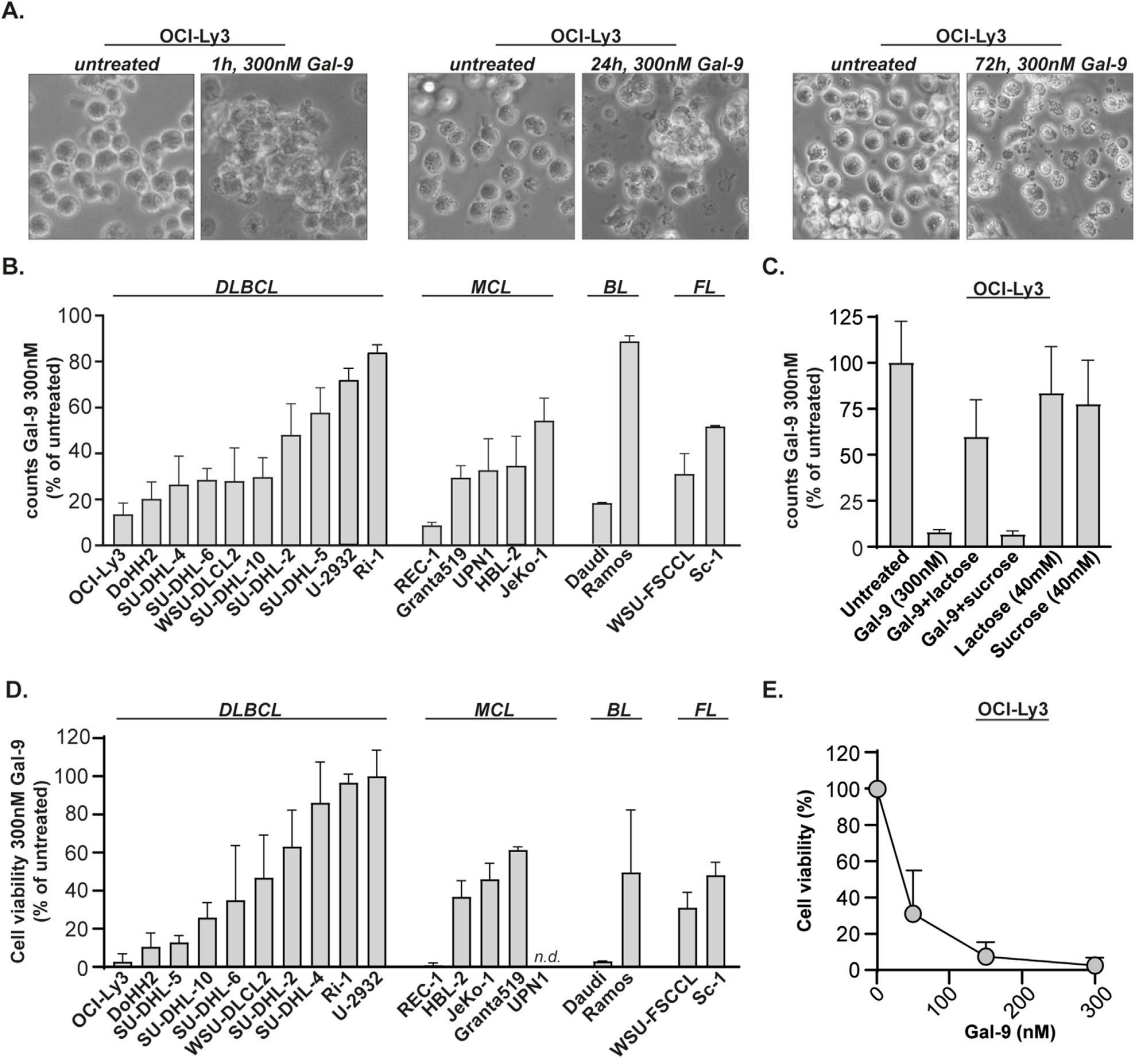
Galectin-9 is cytotoxic for B cell lymphoma cell lines. **(A)** The DLBCL cell line OCI-Ly3 was treated with Gal-9 (300 nM) and brightfield microscopic pictures were taken after 1 h, 24 h and 72 h incubation. **(B)** Counts, depicted as percentage of untreated, for B cell lymphoma cell lines treated with 300 nM Gal-9 for 24 h (n = 3) analyzed with flow cytometry (see Supplementary Figure S1A for counting method). **(C)** As in **(B)**, but in the presence of 40 mM α-lactose (CRD-blocking agent) or 40 mM sucrose (non-CRD binding sugar). **(D)** Cell viability, depicted as percentage of untreated, for B cell lymphoma cell lines treated with 300 nM Gal-9 for 72 h (n = 3) analyzed with the MTS assay. Since the UPN1 cell line was not capable of converting MTS, i.e., no color switch was observed, this cell line was not included in the data. **(E)** Dose-response curve for OCI-Ly3 using the indicated concentrations of Gal-9 and analyzed with the MTS assay (72 h, n = 3).

Gal-9 was proven to be cytotoxic for various types of malignant cells, including melanoma, colon and acute myeloid leukemia (Wiersma et al., 2015; Wiersma et al., 2012; Choukrani et al., 2023). To determine whether Gal-9 also induced cell death in B cell lymphoma, the diffuse large B-cell lymphoma (DLBCL) cell line OCI-Ly3 was treated with a recombinant form of Gal-9 (Gal-9 (0), called Gal-9 hereafter) (Nishi et al., 2005). Treatment with Gal-9 (300 nM) induced aggregation of the cells already after 1 h of incubation (Figure 1A, left panel). After 24 h of treatment with Gal-9 most of the cells had a granular morphology with cell debris being detected, indicative of cell death, which came even more apparent after 72 h of incubation (Figure 1A, middle and right panel). In line with visual observations, treatment with Gal-9 (300 nM, 24 h) decreased total cell counts in a panel of DLBCL, MCL, Burkitt lymphoma (BL) and follicular lymphoma (FL) cell lines (Figure 1B). Of note, these cell counts were taken from the ‘viable gate’ based on FSC/SCC flow cytometry plots, which were indeed negative for PI, a dye that stains leaky cells (Supplementary Figure S1A). The cytotoxic effect of Gal-9 was dependent on its carbohydrate recognition domain (CRD) as co-incubation with the CRD-blocking sugar α-lactose, but not the non-CRD binding sugar sucrose, inhibited the reduction in total cell counts (Figure 1C). In line with the counting data, Gal-9 treatment (300 nM, 72 h) reduced cell viability in almost all cell lines tested (Figure 1D), which was dose dependent (Figure 1E; Supplementary Figure S1B). Of note, there was quite some variation in sensitivity between the cell lines (Supplementary Figure S1B). As expected, viable cell count and MTS data significantly correlated with each other for the majority of the cell lines (Supplementary Figure S1C). Of note, Gal-9(S), one of the natural occurring isoforms of Gal-9, also reduced cell counts (Supplementary Figure S2A) and decreased cell viability (Supplementary Figure S2B). However, Gal-9 cytotoxicity was not restricted to malignant B cells as isolated B cells from healthy donors (∼80% pure, Supplementary Figure S2C) were also affected by Gal-9 treatment (Supplementary Figures S2D–E). Taken together, Gal-9 has direct cytotoxic activity toward various types of B cell lymphoma.

### Galectin-9 inhibits autophagy in sensitive B cell lymphoma lines

**FIGURE 2:**
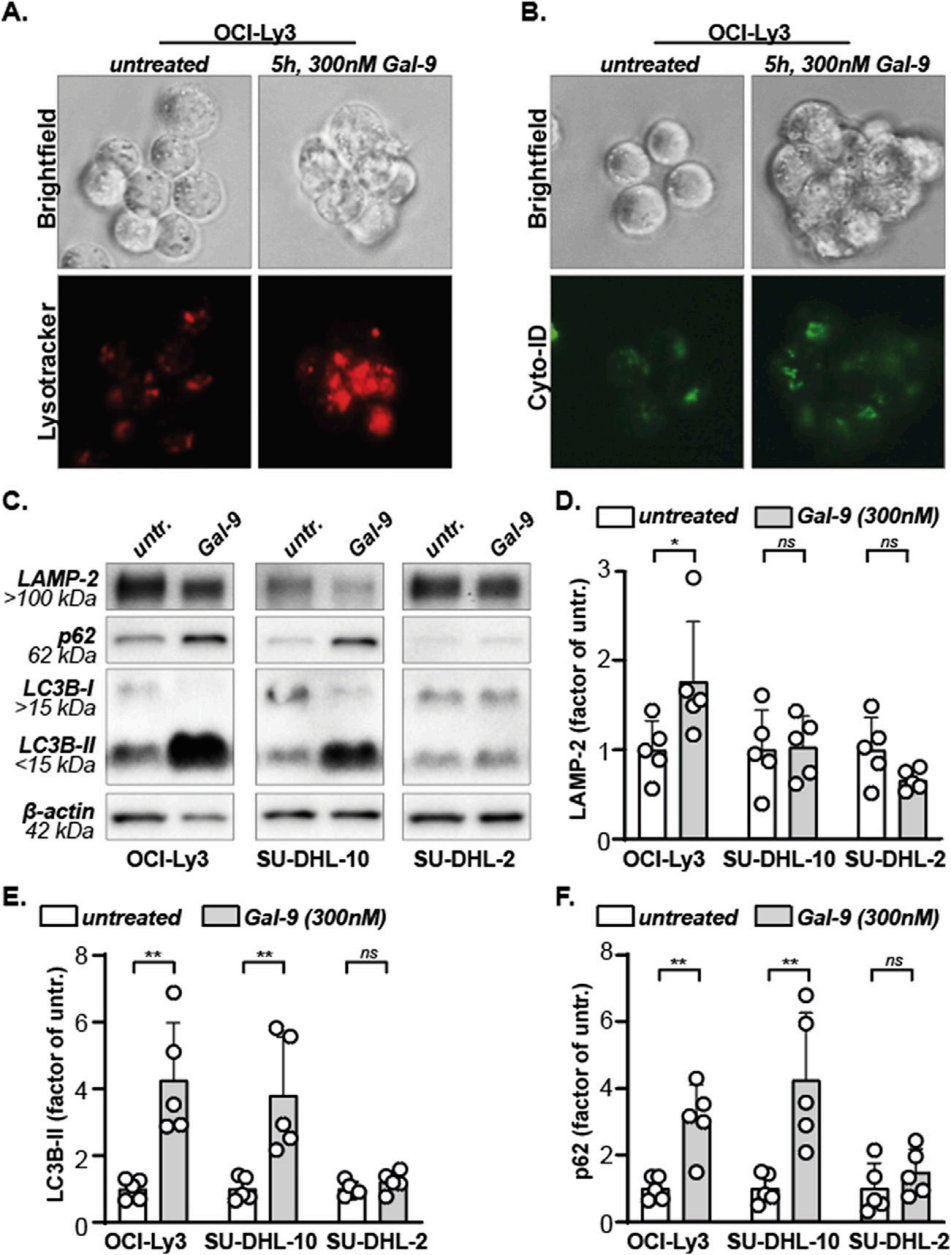
Galectin-9 inhibits autophagy. **(A)** Lysosomes, stained red using lysotracker, in untreated versus Gal-9 treated (5 h, 300 nM) OCI-Ly3 cells. Representative image of three independent experiments. **(B)** Autophagosomes, stained green using Cyto-ID, in untreated versus Gal-9 treated (5 h, 300 nM) OCI-Ly3 cells. Representative image of three independent experiments. **(C)** Expression levels of the depicted proteins with and without Gal-9 treatment (300 nM, 24 h). Representative blot of five independent experiments. **(D)** Analyzed LAMP-2 expression levels, **(E)** LC3B-II expression levels, and **(F)** SQSTM1/p62 expression levels, depicted as factor of the average untreated, for all five independent experiments.

We previously demonstrated that Gal-9 impairs the execution of autophagy in both solid and hematological cancers causing lysosomal swelling and accumulation of autophagosomes (Wiersma et al., 2015; Choukrani et al., 2023). Correspondingly, Gal-9 treatment increased lysotracker and Cyto-ID signals in B cell lymphoma cells, indicative of the involvement of the lysosomal and autophagosomal pathway (Figures 2A,B). To further investigate the impact of Gal-9 on the lysosomal-autophagosomal pathway, protein levels of LAMP-2, p62, LC3B-I and LC3B-II were determined in DLBCL cells upon treatment with Gal-9. To this end, a very sensitive (OCI-Ly3), moderately sensitive (SU-DHL-10) and weakly sensitive (SU-DHL-2) DLBCL cell line was selected (based on data in Figure 1). LAMP-2 is a lysosomal marker, and its expression significantly increased upon Gal-9 treatment in the OCI-ly3 cell line, but not in the SU-DHL-10 or SU-DHL-2 cell line (Figures 2C,D). Gal-9 treatment also clearly increased LC3B-II levels in the most sensitive cell lines (OCI-Ly3, SU-DHL-10), but not in the less sensitive cell line (SU-DHL-2) (Figures 2C,E). Notably, LC3B-I is converted to LC3B-II upon activation of the autophagy pathway, hence an increase in LC3B-II is seen upon autophagy activation (Folkerts et al., 2019). However, this increase should be temporary as LC3B-II is degraded and/or recycled when the lysosomes fuse with the autophagosomes (Ni et al., 2011). Hence prolonged accumulation of LC3B-II, as induced by Gal-9 in the sensitive cell lines, is seen in case the execution phase of autophagy is inhibited. In addition, p62 levels also only accumulated in the two most sensitive cell lines (Figures 2C,F). p62 is a cargo protein that guides the to be degraded cellular content to the autophagosomes. Upon fusion with the lysosomes during the final executional step of autophagy, p62 is degraded as well. Hence, the accumulation of both LC3B-II and p62 upon Gal-9 treatment indicates that the execution of autophagy is inhibited in OCI-Ly3 and SU-DHL-10 cells at the stage of autophagosome-lysosome fusion. Comparable results were obtained for the sensitive cell line Daudi, moderately sensitive cell line Sc-1 and rather resistant cell line U2932 (Supplementary Figure S3A). Together, this data implies that Gal-9 inhibits the proper execution of autophagy in cell lines that are sensitive for this lectin.

### Basal protein expression of LC3B-I associates with Gal-9 sensitivity in B cell lymphoma cells

**FIGURE 3:**
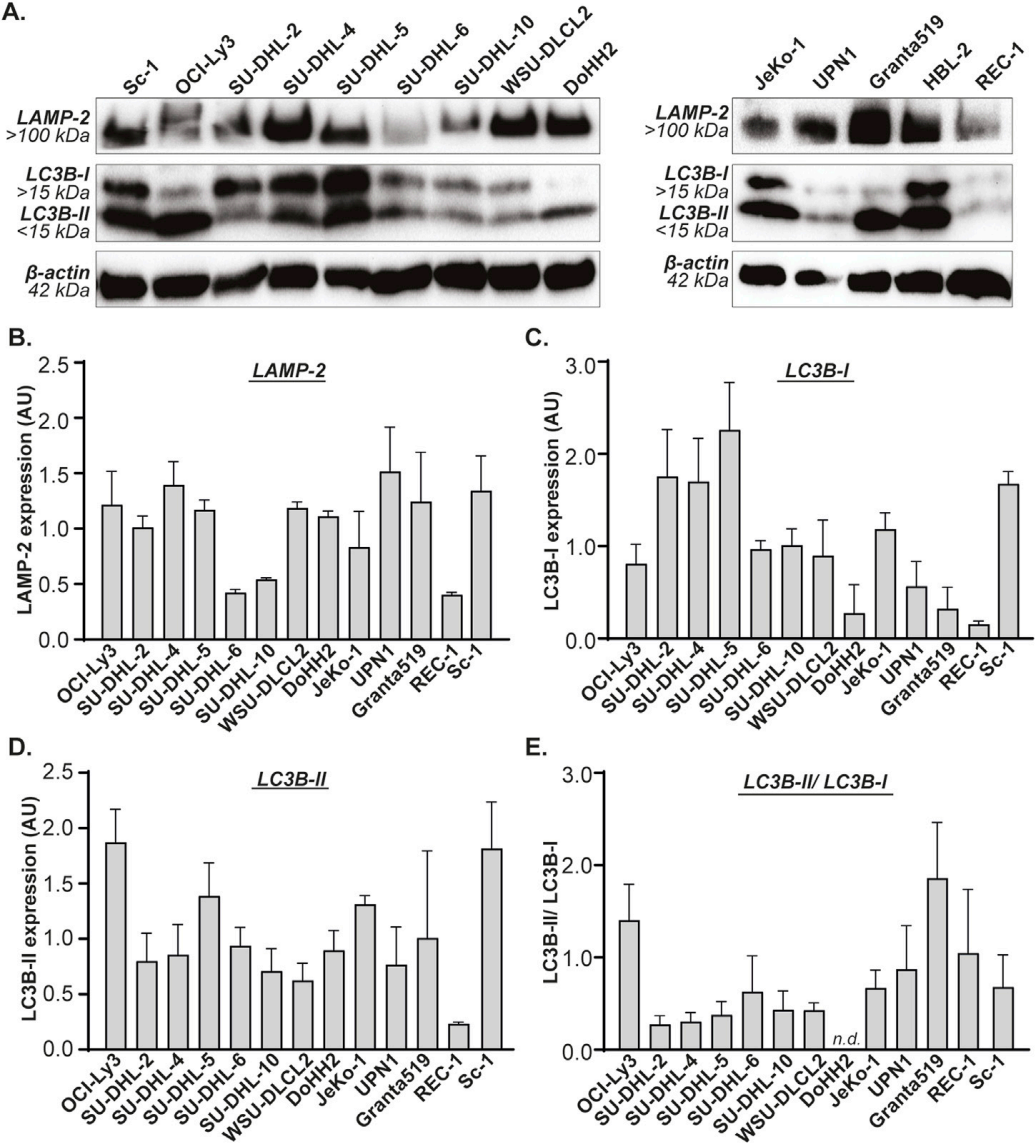
Basal expression levels of LAMP-2 and LC3B in B cell lymphoma cell lines. **(A)** Western blot of B cell lymphoma cell lines, depicting basal expression levels of LAMP-2, LC3B-I and LC3B-II. Quantified expression levels of **(B)** LAMP-2, **(C)** LC3B-I, **(D)** LC3B-II, and **(E)** the LC3B-II/LC3B-I ratio. All n = 2. DoHH2 lacks the LC3B-II/LC3B-I ratio as the LC3B-I protein was undetectable for one of the replicates.

Autophagy is induced during periods of stress, but all cells maintain a basal level of autophagy. To investigate whether Gal-9 sensitivity depends on basal autophagy levels, the expression of prominent players of the autophagy pathway were determined in B cell lymphoma cell lines on both mRNA and protein level. The mRNA expression levels of *LC3B*, *SQSTM1* and *LAMP2* differed greatly between all cell lines (Supplementary Figure S3B-D). This was also reflected by the protein expression levels of their respective proteins, i.e., LC3B-I, LC3B-II, p62 and LAMP-2 (Figures 3A–D), as well as the LC3B-II/LC3B-I ratio (Figure 3E). Similarly, mRNA expression levels of *LAMP1* and *ATG5* were quite variable between cell lines (Supplementary Figures S3E, F).

**FIGURE 4:**
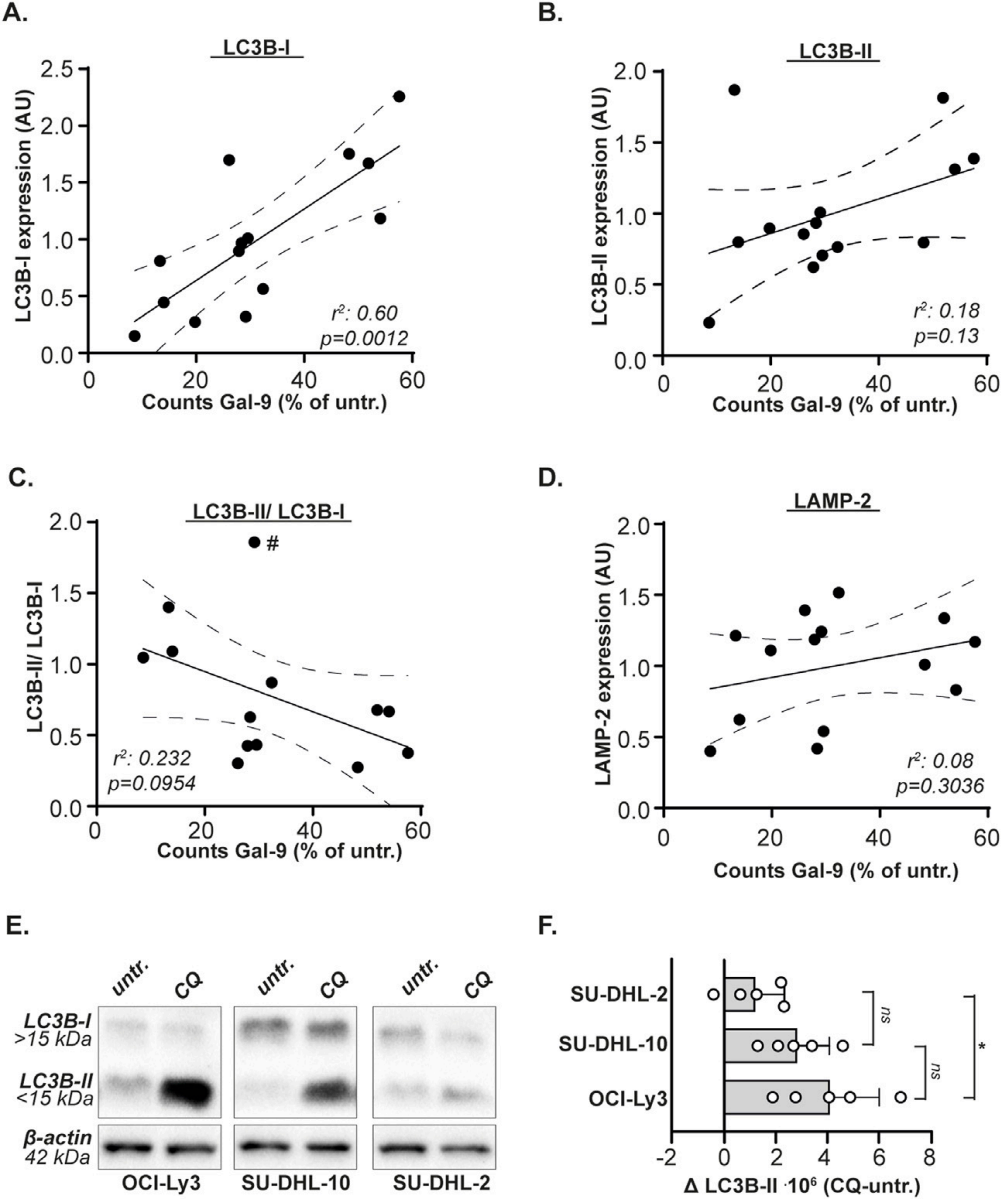
Gal-9 sensitivity depends on LC3B-I protein expression levels and autophagic flux. Correlation between Gal-9 sensitivity, depicted as counts after Gal-9 treatment (see Figure 1C), and protein expression levels (see Figure 3) of **(A)** LC3B-I, **(B)** LC3B-II, **(C)** LC3B-II/LC3B-I, and **(D)** LAMP-2. **(E)** Expression levels of LC3B-I and LC3B-II with and without CQ treatment (50µM, 6 h). Representative blot of five independent experiments. **(F)** Analyzed autophagic flux, calculated as CQ treated-untreated, for the five independent experiments. The # in Figure 4C indicates the Granta519 cell line.

Next, the expression levels of the autophagy genes and proteins were associated with Gal-9 sensitivity (as determined in Figure 1C). The sensitivity for Gal-9 did not significantly associate with mRNA expression levels of any of the analyzed genes (Supplementary Figure S4A). In contrast, the sensitivity of B cell lymphoma cell lines for Gal-9 significantly correlated with basal protein expression levels of LC3B-I, whereby cell lines with lower basal LC3B-I levels were more sensitive toward Gal-9 treatment than cell lines with higher LC3B-I levels (Figure 4A). Also the association between Gal-9 sensitivity and LC3B-II levels or the LC3B-II/LC3B-I ratio demonstrated a trend towards association, although this was not significant (Figures 4B,C). This lack of significance was caused by the Granta519 cell line (indicated with the # in Figure 4C) that had the highest LC3B-II/LC3B-I ratio, but following the correlation a too low sensitivity for Gal-9. When excluding this cell line, the linear regression analysis would be significant (*r* ^2^ = 0.37, p = 0.0347). A higher LC3B-II/LC3B-I ratio would mean that more of this protein has been converted into the active LC3B form, which corresponds to a higher autophagic flux. There was no significant association between basal LAMP-2 protein levels and Gal-9 sensitivity (Figure 4D). Thus, Gal-9 sensitivity associates with basal protein expression of LC3B-I in B cell lymphoma cells, potentially related to basal autophagic flux levels.

### B cell lymphoma cells with high basal levels of autophagic flux are more sensitive to Galectin-9

As demonstrated above, cell lines with lower basal LC3B-I Levels were more sensitive toward Gal-9 treatment than cell lines with higher LC3B-I levels. Less LC3B-I may mean more autophagy, as the protein is actively converted to LC3B-II upon autophagy activation. Especially in combination with the trend of cells being more sensitive to Gal-9 treatment when their LC3B-II/LC3B-1 ratio is higher, this suggests that Gal-9 sensitivity is related to the basal activity of the autophagy pathway. To validate this hypothesis, the very sensitive OCI-Ly3, moderately sensitive SU-DHL-10, and weakly sensitive SU-DHL-2 DLBCL cell line was treated with chloroquine (CQ). CQ is a lysosomotropic agent that accumulates in the lysosomes, thereby inhibiting the execution of autophagy at the level of lysosome-autophagosome fusion. Hence, the accumulation of LC3B-II upon CQ treatment can be used as measure of basal autophagic flux (Zhang et al., 2016; Mizushima et al., 2010). It is evident that the most sensitive cell line OCI-Ly3 accumulated the most LC3B-II (Figures 4E,F), and also the moderately sensitive cell line SU-DHL-10 accumulated more LC3B-II upon CQ treatment compared to the weakly sensitive cell line SU-DHL-2 (Figures 4E,F). Also Daudi, which is equally sensitive to Gal-9 as compared to OCI-Ly3 (Figures 1B,D), demonstrated an equally high autophagic flux and comparable basal LC3B-I levels as OCI-Ly3 in an additional independent analysis (Supplementary Figure S4B). Together, this data suggest that Gal-9 sensitivity of B cell lymphoma cells is related to basal levels of autophagy flux.

### Therapy resistant B cell lymphoma cells can be eradicated using Galectin-9

**FIGURE 5:**
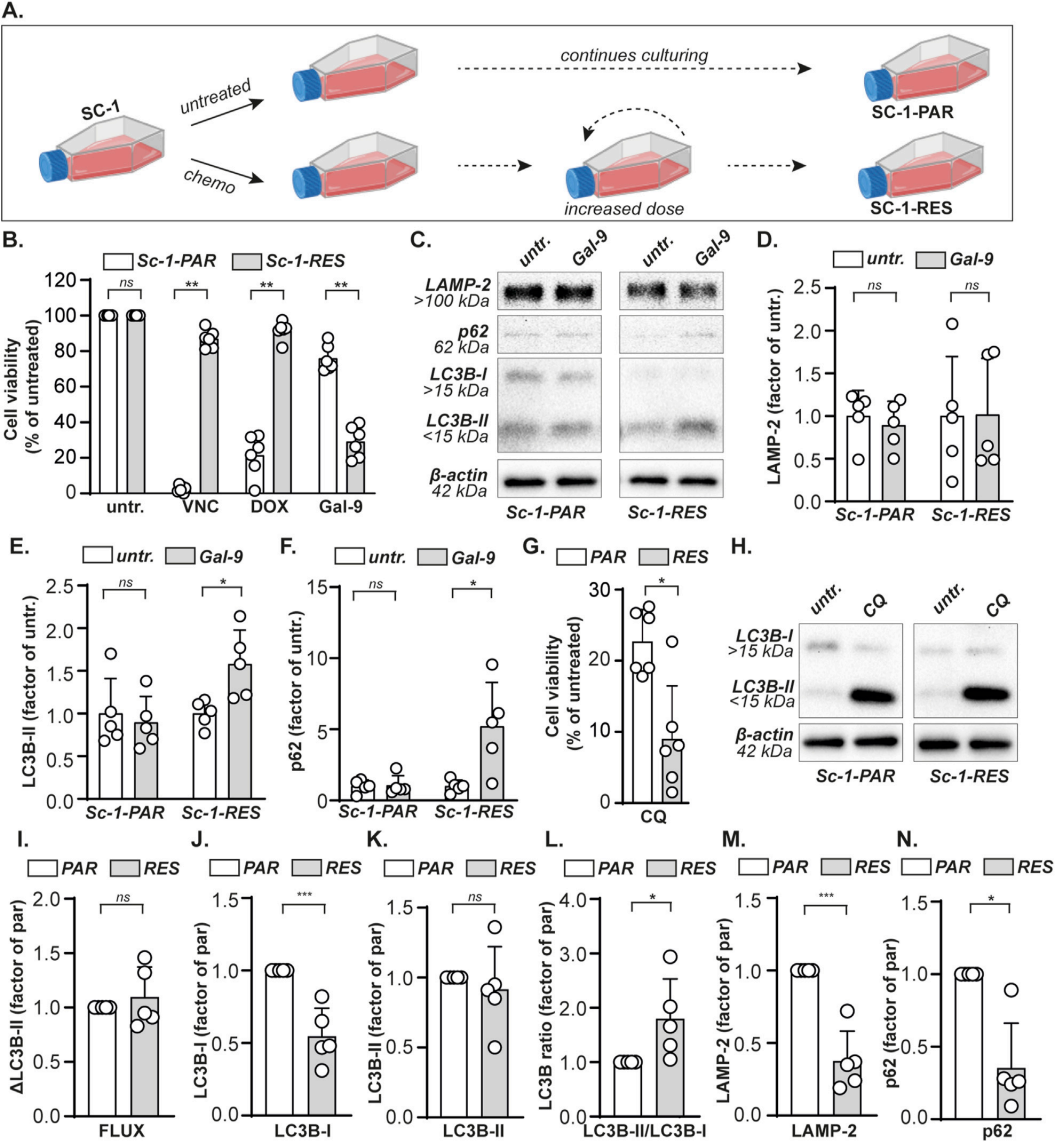
Gal-9 eradicates chemoresistant B cell lymphoma cells. **(A)** A VNC and DOX double resistant Sc-1 cell line was obtained by culturing under gradually increasing concentrations chemotherapy (see material and methods), yielding parental (Sc-1-PAR) and resistant (Sc-1-RES) cells. **(B)** Cell viability determined with the MTS assay, upon treatment with VNC (10 nM), DOX (60 nM) or Gal-9 (300 nM) for 72 h. **(C)** Expression levels of the depicted proteins with and without Gal-9 treatment (300 nM, 24 h). Representative blot of five independent experiments. Quantified expression levels of **(D)** LAMP-2, **(E)** LC3B-II, and **(F)** p62 in Gal-9-treated Sc-1-PAR and Sc-1-RES cell lines of the western blots (n = 5) as depicted in **(C)** normalized as factor of untreated. **(G)** Cell viability upon treatment with CQ (50 µM) for 72 h, as determined by the MTS assay. **(H)** LC3B-I and LC3B-II levels with and without CQ treatment (50µM, 6 h). Representative blot of five independent experiments. Quantified basal expression levels, depicted as factor between PAR and RES (whereby PAR = 1), of **(I)** the autophagic flux **(J)** LC3B-I, **(K)** LC3B-II, **(L)** the LC3B-II/LC3B-I ratio, **(M)** LAMP-2, and **(N)** p62 in Sc-1-PAR and Sc-1-RES cell lines of the western blots (n = 5) as depicted in **(C, H)**.

To investigate the sensitivity of chemoresistant B cell lymphoma towards Gal-9 treatment, Vincristine (VNC) and Doxurubicin (DOX) resistant Sc-1 cells were generated by culturing under constant pressure of increasing doses of chemotherapy (Figure 5A), resulting in the cell line panel: Sc-1-parental (Sc-1-PAR) and Sc-1-resistant (Sc-1-RES). Indeed, both VNC and DOX did not reduce the cell viability of Sc-1-RES cells (Figure 5B), also not in combination (cultured under constant pressure of 10 nM VNC and 60 nM DOX). Interestingly, Gal-9 remained cytotoxic for Sc-1-RES cells, which even significantly gained in sensitivity compared to Sc-1-PAR cells (Figure 5B). LAMP-2 levels did not change upon Gal-9 treatment (Figures 5C,D), whereas the accumulation of p62 and LC3B-II was significantly higher in Sc-1-RES cells as compared to Sc-1-PAR cells (Figures 5C,E,F). Furthermore, SC1-RES cells were more sensitive toward CQ treatment as compared to Sc-1-PAR (Figure 5G), although the basal autophagic flux based on CQ-induced LC3B-II in the Sc-1-RES cells was not significantly elevated (Figures 5H,I). However, in line with the data of the cell line panel, basal protein expression levels of LC3B-I were significantly lower in the more Gal-9-sensitive Sc-1-RES cell line (Figures 5C,H,J). Further, basal LC3B-II protein levels did not differ (Figures 5C,H,K), whereas the LC3B-II/LC3B-I ratio was higher in the Sc-1-RES cell line (Figure 5L). Also both basal expression levels of LAMP-2 and p62 protein were significantly lower in the Sc-1-RES cell line compared to Sc-1-PAR (Figures 5M,N). Thus, although the autophagic flux as calculated by CQ-induced LC3B-II levels did not differ between Sc-1-PAR and Sc-1-RES, the lower LC3B-I levels, higher LC3B-II/LC3B-I ratio, increased CQ sensitivity, and lower basal p62 levels do all suggest that the Sc-1-RES cells have a higher autophagic turnover. Together, Gal-9 is cytotoxic for B cell lymphoma cells, also when chemoresistant, by inhibiting the proper execution of autophagy.

## Discussion

In this study it was demonstrated that Gal-9 is cytotoxic for a variety of human B cell lymphoma cell lines, including a chemoresistant one. Mechanistically, Gal-9 inhibited the proper execution of autophagy, whereby cells with lower basal expression levels of LC3B-I protein were more sensitive towards this lectin. Thus, Gal-9 is a potent inducer or cell death in (chemoresistant) B cell lymphoma.

The ability of Gal-9 to inhibit the autophagy pathway may be of especial interest since cancer cells commonly upregulate this pathway. Indeed, a stronger LC3B staining was observed in biopsies of aggressive DLBCL compared to indolent lymphomas, suggesting that lymphoma cells are highly autophagy-dependent (Mandhair et al., 2024). The autophagy pathway is stimulated during periods of stress, as induced by chemotherapy. The primary line of treatment for B cell lymphoma is R-CHOP, and both doxorubicin (Chen et al., 2018) and vincristine (Hsieh et al., 2015) are known to activate autophagy. However, the Sc-1-RES cell line did not have a higher basal autophagic flux as compared to the Sc-1-PAR cell line. This finding is in line with our previous study in which already established cytarabine-resistance did not result in a higher basal autophagic flux in AML cells, whereas autophagy was induced upon cytarabine treatment of sensitive cells (Visser et al., 2021). However, as protein levels of LC3B-I, LAMP-2 and p62 were significantly lower in the Sc-1-RES cells compared to Sc-1-PAR, and a higher LC3B-II/LC3B-I ratio was observed in Sc-1-RES, this may still suggest a higher turn-over of lysosomes and autophagosomes in these chemoresistant cells. Further in-depth analysis of autophagy, for instance by using LC3-GFP-mcherry reporter constructs (Wiersma et al., 2015; Choukrani et al., 2023), may shed more light on the involvement of this pathway.

Notably, lymphoma cells can escape from apoptotic cell death by upregulating anti-apoptotic proteins of the Bcl family (Bernal-Mizrachi et al., 2006; Johnson et al., 1993) or loss of pro-apoptotic proteins like Bax (Diepstraten et al., 2023). Therefore, targeting the autophagy pathway may be a promising way to eradicate (apoptosis-resistant) lymphoma cells. Indeed, the artemisinin derivative SM1044 induced autophagy-dependent cell death in DLBCL (Cheng et al., 2018). Similarly, targeting the autophagy pathway by inhibiting ULK1, a protein important for the initiation of autophagy, reduced the viability of DLBCL cells (Mandhair et al., 2024). Also in the current study, both Sc-1-PAR and Sc-1-RES cells were sensitive for the autophagy inhibitors CQ and Gal-9, whereby both agents had the strongest impact on Sc-1-RES cells. Thus, the inhibition of the proper execution of autophagy, as induced by Gal-9, may be a promising approach to eradicate (chemoresistant) DLBCL.

Both malignant and healthy B cells were sensitive toward Gal-9 treatment. In light of clinical application this is acceptable since current chemotherapy regimen also do not spare healthy B cells, and B cells are commonly repopulated within 1 year after therapy (Ito et al., 2016; Anolik et al., 2007). Also in T cells, Gal-9 was cytotoxic for both malignant and healthy cells (Gooden et al., 2013; Lhuillier et al., 2015; Lu et al., 2007; Kashio et al., 2003), which was dose dependent as lower Gal-9 concentrations could also induce T cell proliferation (Gooden et al., 2013). This is in contrast to melanoma (Wiersma et al., 2012), colon carcinoma (Wiersma et al., 2015), and CD34^+^ AML stem cells (Choukrani et al., 2023), where Gal-9 had no effect towards their healthy counterparts. Whether Gal-9 is only cytotoxic for malignant cells or also healthy cells likely depends on the glycan patterns present on these cells. Gal-9 is a lectin that recognizes β-galactosides, having a strong binding toward poly-N-acetyllactosamine (poly-LacNAc). Healthy B cells, including naive, germinal center, and memory B cells, express high levels of poly-LacNAc (Giovannone et al., 2018), hence Gal-9 can bind to them and induce cell death, like in lymphoma cells. However, poly-LacNAc expression has been described to be increased in solid cancers as compared to healthy cells and correlate with disease progression, in among others melanoma (Kinoshita et al., 2014) breast cancer (Scott et al., 2019) and colon cancer (Ishida et al., 2005). Correspondingly, Gal-9 was only cytotoxic for malignant colon carcinoma and melanoma cells, and not their healthy counterparts (Wiersma et al., 2015; Wiersma et al., 2012). Therefore, although not formally proven yet, specific sensitivity of malignant cells for Gal-9 treatment, or the lack hereof, may rely on glycan expression patterns. In line with this, the induction of B cell lymphoma cell death by Galectin-1 and Galectin-3 also depended on the surface glycosylation of these cells (Suzuki et al., 2005; Suzuki and Abe, 2008).

Differences in sensitivity may also rely on the routing of internalized Gal-9, as Gal-9 was recycled back to the apical surface in polarized non-malignant MDCK cells (Mishra et al., 2010), but transported to and accumulated in lysosomes in cancer cells (Wiersma et al., 2015; Choukrani et al., 2023; Itoh et al., 2019). Of note, the localization of Gal-9 at the lysosomal compartments has been reported to depend on its interaction with LAMP-2 (Sudhakar et al., 2020). This lysosomal localization of Gal-9 was required for maintaining homeostatic function of lysosomes. Furthermore, galectins were found to control autophagy upon lysosomal damage (Jia et al., 2018), whereby Gal-9 was responsible for the activation of AMP-activated protein kinase, a positive regulator of autophagy. In addition, Gal-9 localized at autophagosomes during the degradation of its interacting proteins (Miyakawa et al., 2022; Wang et al., 2021). Therefore, the effect of Gal-9 on the execution of autophagy may stem from its ability to interact with and modulate the function of autophagosomes and lysosomes. Thus, it seems that Gal-9 is required for maintaining lysosomal and autophagosomal functioning, but on the other hand impairs their fusion when used as therapeutic agent. This discrepancy likely depends on the difference between endogenously expressed, and exogenously added Gal-9, which predominantly differ in concentration. Specifically, endogenous serum Gal-9 levels range from 1,5 to 10 ng/ml (=45–300 pM) in healthy individuals (Chen et al., 2024; Konantz et al., 2023; Shih et al., 2024; He et al., 2017; Wdowiak et al., 2019; Wiersma et al., 2019), whereas 300 nM was used in this study. Hence, low (endogenous) concentrations of Gal-9 may be beneficial for maintaining the autophagy pathway and/or cell survival, whereas high (exogenous) concentrations inhibit the execution of autophagy, promoting cell dead. In this respect, endogenous Gal-9 has also been reported as driver of AML stem cells (Kikushige et al., 2015), and its inhibition is currently being tested in clinical trials [NCT05829226, NCT04666688]. Thus, high levels of exogenously added Gal-9, as used in this study, induce cancer cell death by inhibiting autophagy, whereas lower endogenous Gal-9 levels may have opposite effects.

Furthermore, the expression of Gal-9 binding partners likely play an important role in dictating sensitivity towards this lectin. Endogenous Gal-9 (Giovannone et al., 2018) as well as exogenous Gal-9 (Cao et al., 2018) could be detected on the surface of healthy B cells, suggesting the presence of interaction partners on the plasma membrane. Also on DLBCL cells endogenous Gal-9 could be detected (Henry et al., 2022). Interaction partners of Gal-9 on B cells were not determined in the current study, but previous pull-down studies identified several Gal-9 binding partners including CD45 and IgM-B cell receptor (BCR) on primary B cells (Cao et al., 2018). The interaction of recombinant Gal-9 with the BCR altered the organization of CD45 and CD22, thereby suppressing BCR-mediated B cell signaling. This finding was also found by another independent study, that demonstrated that Gal-9 binds to CD45 to trigger inhibitory signaling via CD22 and downstream inhibitory molecules (Giovannone et al., 2018). Interestingly, BCR-signaling is essential for the survival of B cell lymphoma cells, especially for the more aggressive activated B cell like DLBCL subtype (Davis et al., 2010; Young et al., 2015). Therefore, although not investigated in this study, the cytotoxic effect of Gal-9 may also (partly) rely on the inhibition of BCR signaling. However, the Gal-9 concentrations that were used to inhibit BCR-signaling in above mentioned studies, i.e. 15-120 nM Gal-9 (Giovannone et al., 2018) or even up to 1 µM (Cao et al., 2018), are in the same range in which both the natural occurring isoform Gal-9(s) as well as recombinant Gal-9 (0) are cytotoxic for healthy and malignant B cells (Supplementary Figure S2). Therefore, it is debatable whether Gal-9 treatment truly impaired BCR-signaling of B cells in above mentioned studies, or whether the Gal-9-treated B cells displayed reduced BCR-signaling because they were dying. Of note, the expression of Gal-9 interacting proteins *per se* may not be sufficient to induce Gal-9 binding, since they also have to carry the sugar moieties that are recognized by this lectin (as discussed above). In conclusion, Gal-9 is a potent inducer of B cell lymphoma cell dead by inhibiting the proper execution of autophagy. However, further studies are warranted to determine the Gal-9 binding partner(s) and exact mechanisms responsible for the induction of cell death in B cell lymphoma.

## Data Availability

The original contributions presented in the study are included in the article/Supplementary Material, further inquiries can be directed to the corresponding author.
