# Supplementary material for: Galectin-9 treatment is cytotoxic for B cell lymphoma by disrupting autophagy
Source: Front Pharmacol. 2025 Jun 26;16:1601235. doi: 10.3389/fphar.2025.1601235 (PMC12241159; doi:10.3389/fphar.2025.1601235)

Supplementary Figure legends

Suppl. Figure 1: Dose response curves of Gal-9 of all tested B cell lymphoma cell lines. A) Explanation of the cell counting method using the flow cytometer. Based on the FSC/ SCC plots the ‘viable gate’ could be clearly distinguished. That these cells were indeed viable was demonstrated using PI, a dye that only stains leaky cells. Indeed, the cells in the ‘viable gate’ were all PI-negative, whereas the debris in the ‘dead gate’ were all PI-positive, and increased upon Gal-9 treatment. **B)** Cells were treated with indicated doses Gal-9 (50, 150, 300nM) for 72h, and cell viability was assessed using the MTS assay (n=3). Untreated control was set at 100% cell viability. **C)** Correlation between Gal-9 sensitivity, plotting total cell counts (Figure 1C) versus cell viability (Figure 1D), after treatment with 300nM Gal-9.

Suppl. Figure 2: The effect of Gal-9(s) on DLBCL and the effect of Gal-9(0) on healthy B cells. A) Assessment of cell counts after treatment of lymphoma cell lines with Gal-9(0) or Gal-9(s) for 24h. **B)** Cell viability, using the MTS assay, of lymphoma cells upon treatment with Gal-9(0) or Gal-9(s) for 72h. **C)** Purity of B cells after MACS sorting as determined by flow cytometry using an anti-CD19 staining, and gated withing the lymphocyte population based on FSC/ SCC. **D)** Brightfield microscopic pictures of healthy B cells treated with Gal-9 for 24h. **E)** Assessment of cell counts within the viable gate after treatment of lymphoma cell lines with Gal-9 for 24h using flow cytometry.

Suppl. Figure 3: Autophagy inhibition and mRNA expression. A) Expression levels of the depicted proteins with and without Gal-9 treatment (300nM, 24h). Representative blot of five independent experiments. Importantly, these blots were generated independently of the data in Figure 3-4, hence direct comparisons cannot be made. Of note, the Daudi cell line is known to express very low/ undetectable p62 levels, see datasheet of p62 ELISA kit #7814, Cell signaling technology). **B-F)** Basal mRNA expression levels for the depicted genes in the B cell lymphoma cell line panel as determined by RTqPCR.

Suppl. Figure 4: Correlation analysis between Gal-9 sensitivity and mRNA levels. A) Correlation between Gal-9 sensitivity, depicted as counts after Gal-9 treatment (see Figure 1C), and mRNA expression levels (Suppl. Figure 3B-F). **B)** Comparison between autophagic flux and basal LC3B-I levels between OCI-Ly3 and Daudi, two cell lines equally sensitive for Gal-9. Of note, these additional experiments were performed independently of the experiments in Figure 3-4, hence direct comparisons cannot be made.

Suppl. Figure 1

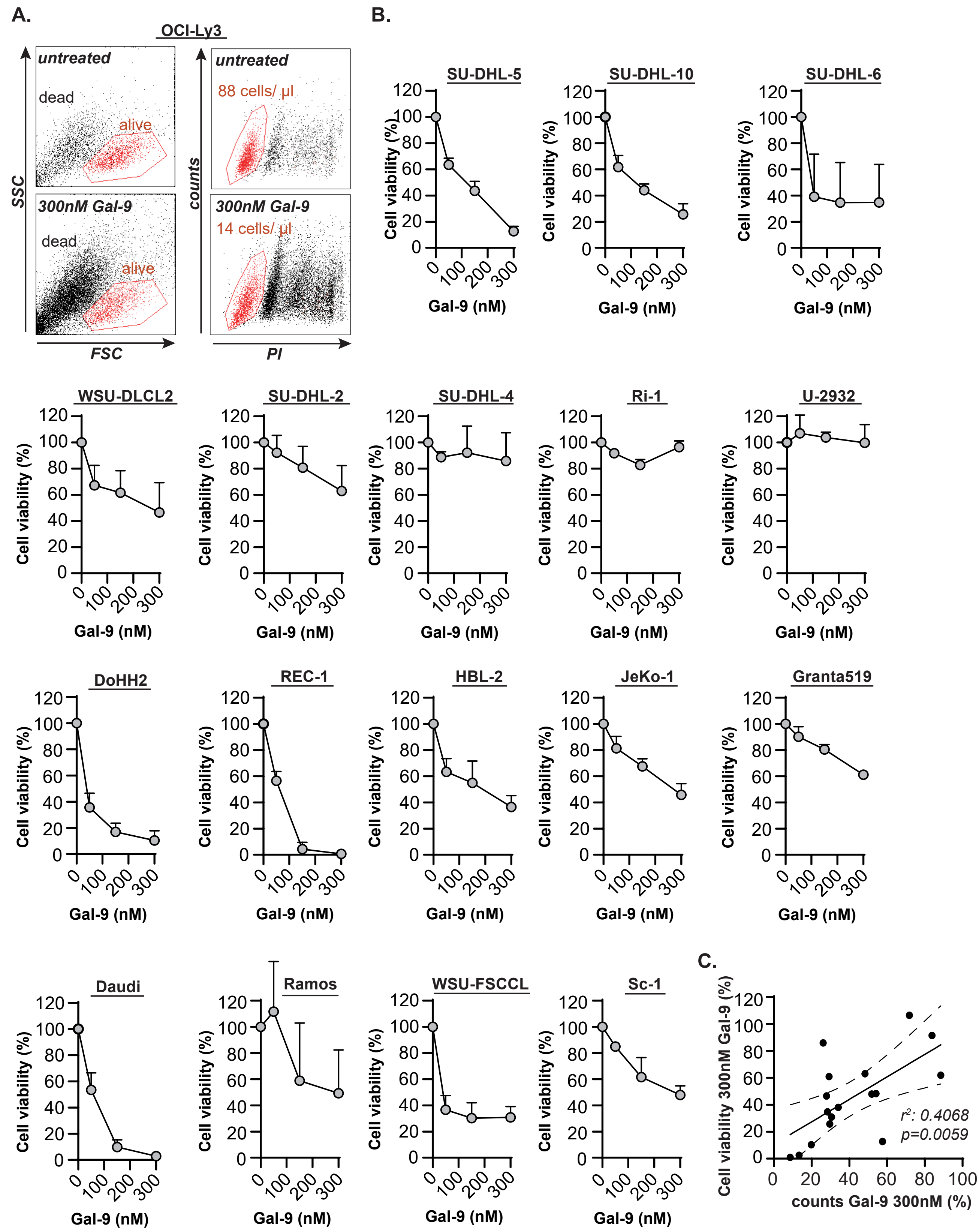

Suppl. Figure 2

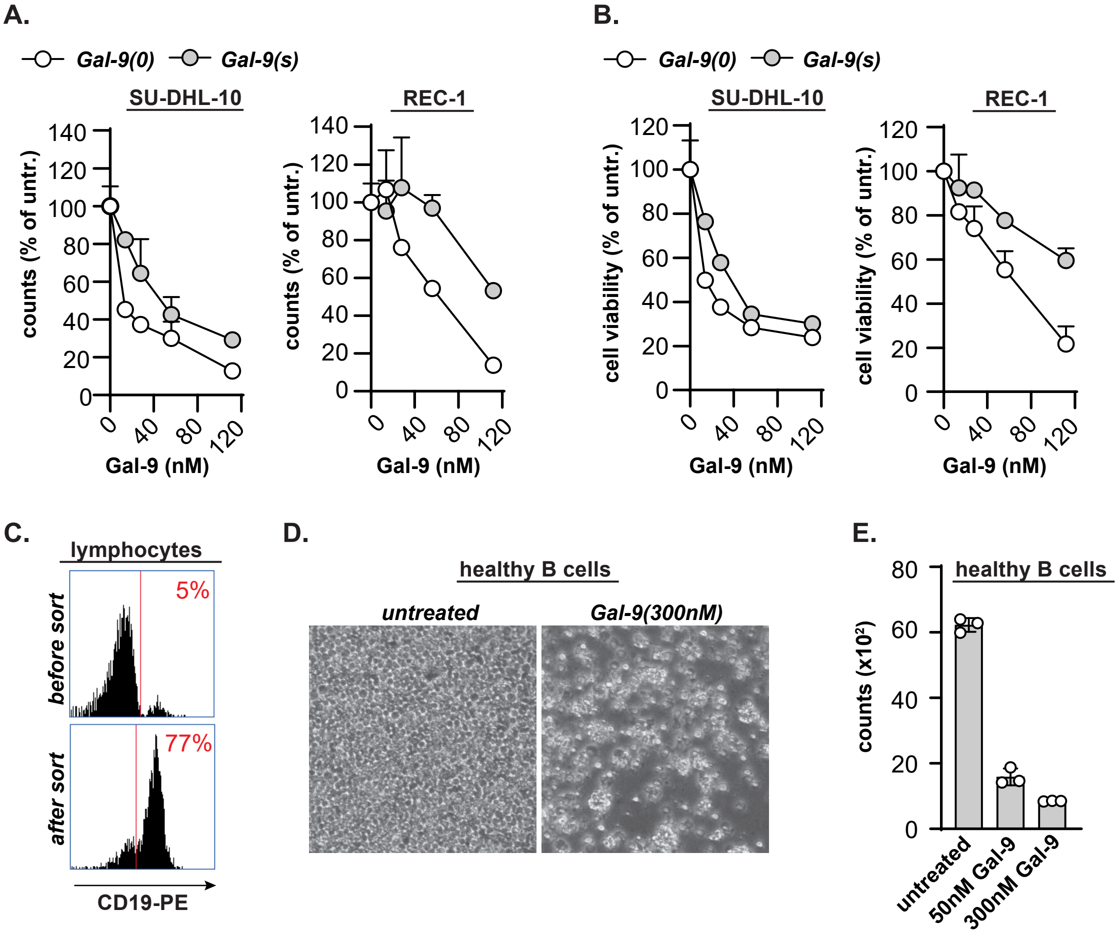

Suppl. Figure 3

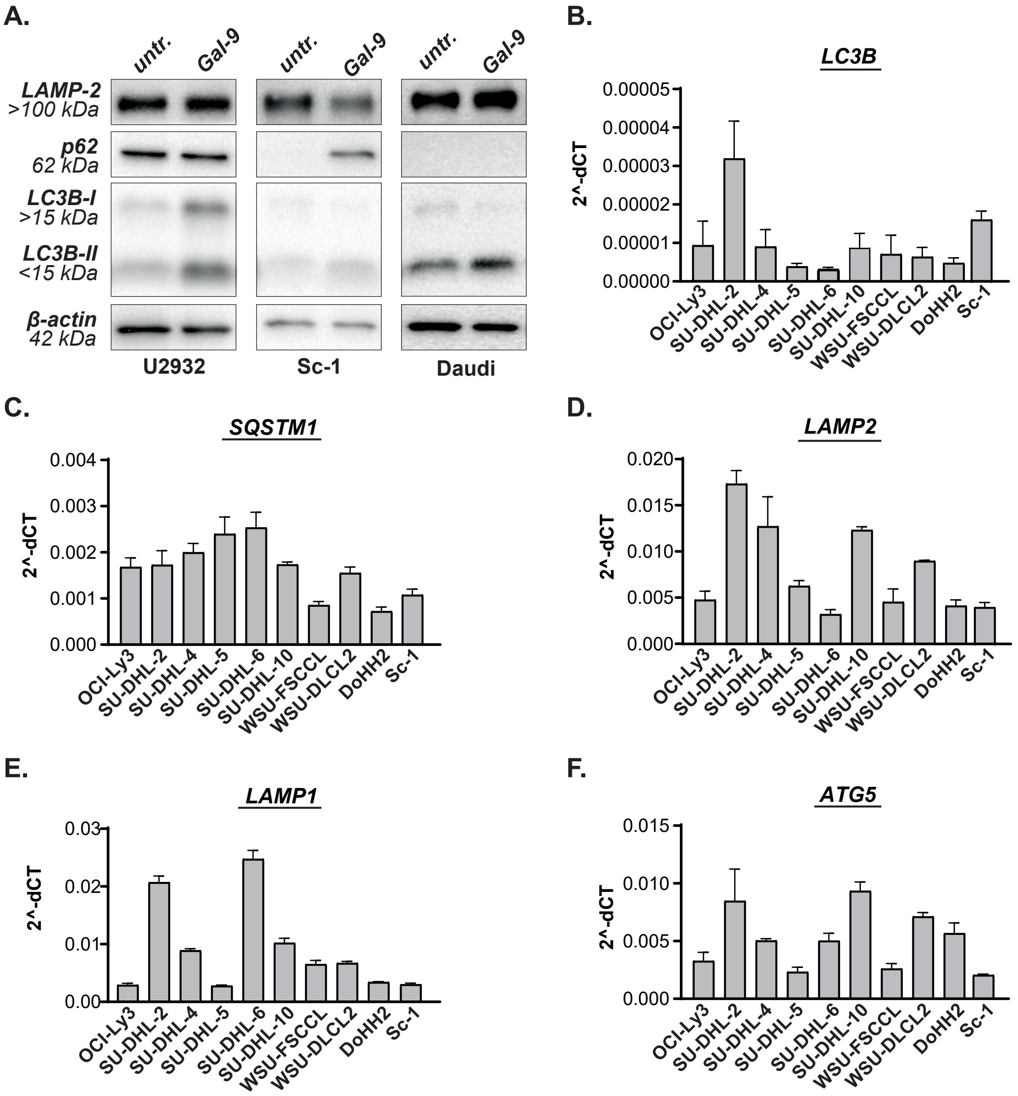

Suppl. Figure 4

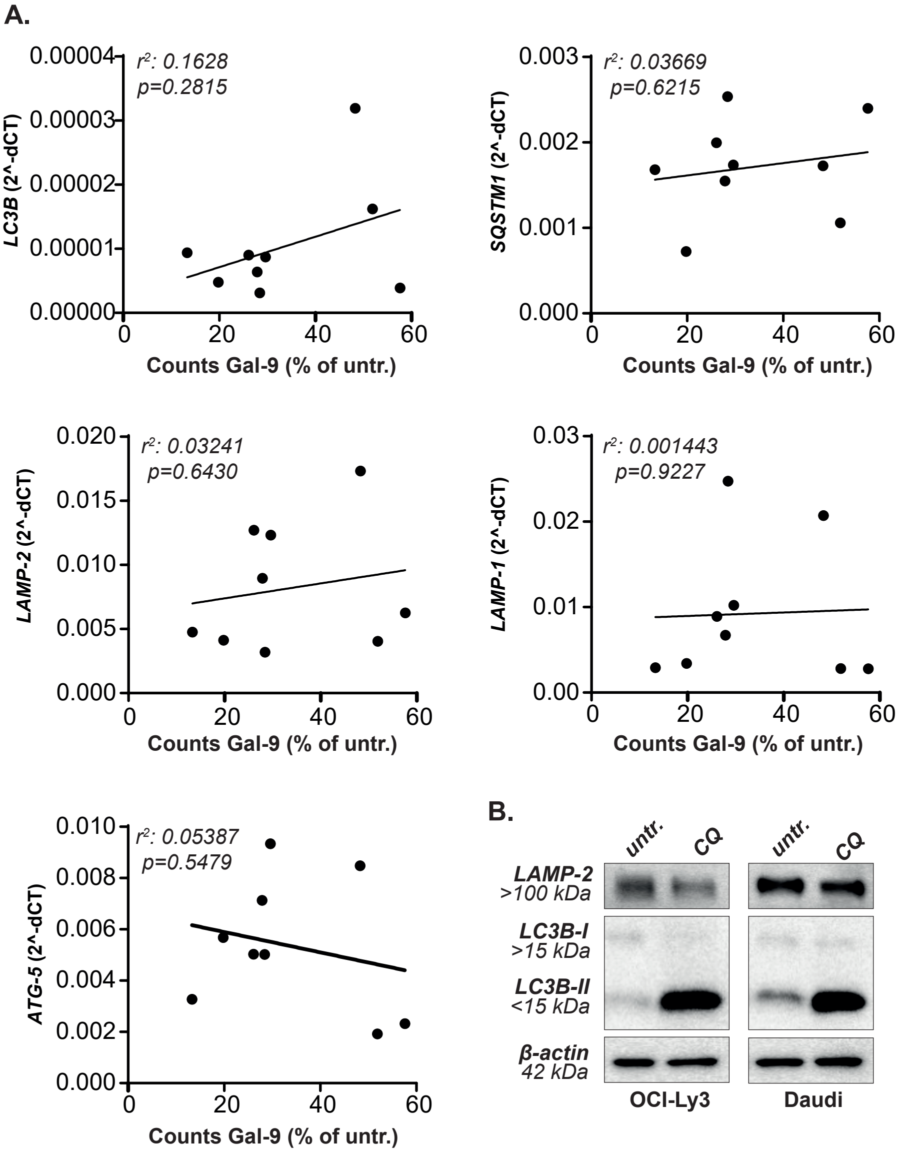

Supplement: Supplementary file 1 [file DataSheet1.pdf]
